# De-DUFing the DUFs: Deciphering distant evolutionary relationships of Domains of Unknown Function using sensitive homology detection methods

**DOI:** 10.1186/s13062-015-0069-2

**Published:** 2015-07-31

**Authors:** Richa Mudgal, Sankaran Sandhya, Nagasuma Chandra, Narayanaswamy Srinivasan

**Affiliations:** IISc Mathematics Initiative, Indian Institute of Science, Bangalore, 560 012 India; Molecular Biophysics Unit, Indian Institute of Science, Bangalore, 560 012 India; Department of Biochemistry, Indian Institute of Science, Bangalore, 560 012 India

**Keywords:** Domain of Unknown Function (DUF), Fold assignment, Function annotation, Remote similarity, Homology detection and protein evolution

## Abstract

**Background:**

In the post-genomic era where sequences are being determined at a rapid rate, we are highly reliant on computational methods for their tentative biochemical characterization. The Pfam database currently contains 3,786 families corresponding to “Domains of Unknown Function” (DUF) or “Uncharacterized Protein Family” (UPF), of which 3,087 families have no reported three-dimensional structure, constituting almost one-fourth of the known protein families in search for both structure and function.

**Results:**

We applied a ‘computational structural genomics’ approach using five state-of-the-art remote similarity detection methods to detect the relationship between uncharacterized DUFs and domain families of known structures. The association with a structural domain family could serve as a start point in elucidating the function of a DUF. Amongst these five methods, searches in SCOP-NrichD database have been applied for the first time. Predictions were classified into high, medium and low- confidence based on the consensus of results from various approaches and also annotated with enzyme and Gene ontology terms. 614 uncharacterized DUFs could be associated with a known structural domain, of which high confidence predictions, involving at least four methods, were made for 54 families. These structure-function relationships for the 614 DUF families can be accessed on-line at http://proline.biochem.iisc.ernet.in/RHD_DUFS/. For potential enzymes in this set, we assessed their compatibility with the associated fold and performed detailed structural and functional annotation by examining alignments and extent of conservation of functional residues. Detailed discussion is provided for interesting assignments for DUF3050, DUF1636, DUF1572, DUF2092 and DUF659.

**Conclusions:**

This study provides insights into the structure and potential function for nearly 20 % of the DUFs. Use of different computational approaches enables us to reliably recognize distant relationships, especially when they converge to a common assignment because the methods are often complementary. We observe that while pointers to the structural domain can offer the right clues to the function of a protein, recognition of its precise functional role is still ‘non-trivial’ with many DUF domains conserving only some of the critical residues. It is not clear whether these are functional vestiges or instances involving alternate substrates and interacting partners.

**Reviewers:**

This article was reviewed by Drs Eugene Koonin, Frank Eisenhaber and Srikrishna Subramanian.

**Electronic supplementary material:**

The online version of this article (doi:10.1186/s13062-015-0069-2) contains supplementary material, which is available to authorized users.

## Background

With the advent of high throughput genomic and proteomic sequencing techniques, we are witnessing a tremendous growth in the sizes of biological sequence databases. However for a large proportion of these putative proteins, structure and function annotation remains either unknown or obscure [1]. Despite enormous improvements in 3-D structure determination methods and co-ordinated efforts on determining structures for proteins with unknown functions [2], the gap between known sequences and their function is widening. Often, grouping of protein sequences into families using sequence, structural or functional similarity, can help in their functional annotation. Clustering proteins into families can aid in identifying domain components, functional motifs, structurally and functionally conserved residues and in appreciating species and sequence divergence.

Pfam database [3] provides one such collection of protein families that are formed on the basis of domain sequence similarity, each represented by multiple sequence alignments and hidden Markov models (HMMs). The Pfam database (version 27.0) includes 14,831 families of which almost 25 % (3,786 out of 14,831) is populated by Domains of Unknown Functions (DUF) and Uncharacterized Protein Families (UPF), both referred to as DUF families henceforth. 699 of the DUF families have at least one member with available structure that can likely provide some clue on protein function, although not necessarily so. For 3,087 Pfam protein families, structure and function is, as yet, unavailable. While 1,421 families are ubiquitous in distribution, 31 % of the DUF families belong only to bacterial species (942 families) and 24 % are exclusive to eukaryotes (724 families). The functional annotation of such domain families, whether commonly shared between or exclusive to genomes, bears significance since they are likely indispensable for the survival of the organism [4]. As and when the functional characterizations of one or more proteins in a DUF family becomes available in the Pfam database, the DUF family is appropriately re-named or merged with the Pfam family with annotated function. In the Pfam database (version 27.0), 303 DUF families were renamed or merged with existing Pfam families. The annotation of existing DUFs seldom keeps pace with new DUFs being added. As a case in point, Pfam database version 27.0 has 242 new DUFs. Regular and periodical assessment of protein annotations through searches in improved databases and through the use of powerful search methods is a worthwhile exercise towards improved protein annotation.

Pfam Clan information in Pfam database provides a valuable resource as it relates families using profile information [5]. Similarity of DUF families with other families of known function or crystal structure can provide important clues, especially since very little is known about their potential biological roles. The association of DUFs with known clans is, therefore, significant and nearly 321 DUF families appear to be already related to a Pfam clan. There remain a large number of DUF families (2,766 out of 3,087) that do not show any significant similarity with other Pfam families. The rapid increase of DUFs with no related functional domains necessitates a comprehensive approach for their structural and functional annotations.

In the absence of any information for these DUF families, experimentally characterizing all the DUF families is intractable. There are numerous challenges in predicting functions from structures; however experimental structure determination or reliable ideas about the structure of proteins in such families can provide valuable insights about the plausible functions and at least help in focusing experimental efforts [6, 7]. Structural information can aid in structure-based detection of distant relatives, active site or ligand-binding site information, putative interfaces for protein-protein interaction, or possible oligomeric states [8–10]. Concerted structural genomics efforts have been carried out across different research groups to solve structures for strategically chosen members from DUF families and to bridge the gap between known sequences and their function [2]. These programs demonstrate that about two-thirds of the 248 structures solved for DUF families show significant structural similarity with known folds, and about one-third of the remaining show significant sub-structure similarity [2]. Although these families are a rich resource for identifying novel folds and functions, any resemblance to known folds can help us formulate hypothesis about their biochemical functions [11]. Domain assignments have, therefore, become an effective starting point for studying and understanding molecular functions. In the recent years, significant improvements and developments have been made in the detection of distant protein similarities through methods based on iterative sequence-profile based searches [12, 13], intermediate sequence-based searches [14, 15] or profile-profile alignment based methods [16–21]. Here we present a ‘computational structural genomics’ approach, where five powerful and sensitive methods and enhanced databases are used to determine distant evolutionary relationships for DUF families. Specifically, we systematically queried profiles or representative sequences from each of the 3,087 DUF families against five different databases, namely SCOP-NrichD database [22, 23], SUPFAM+ database [24], SUPERFAMILY database [25], protein fold library queried using pDomTHREADER [26] and HMM library derived for Structural Classification Of Proteins (SCOP) families [27] using HHsearch [19]. The approach developed in the present study is an amalgamation of searches in enriched databases derived from proteins with known structure and sensitive remote similarity detection algorithms. This approach leads us to identify structural domains and putative functions for about 20 % of the families with no structural or functional information, in essence, De-DUFing the DUFs and, in many cases, can also lead to the identification of critical amino acid residues responsible for protein function.

## Results and discussion

### Assessment of computational methods employed in the study

With the growing disparity between the number of available protein sequences and number of experimentally determined structures and functions, we are reliant on more sensitive and effective computational methods for domain assignments. Over the past decade, highly sensitive methods have been developed to detect weak signals between proteins to classify and annotate them, although with the risk of incorrect assignments and false positives. Therefore, to assess the parameters employed in the searches performed in this study, we compared the domain assignments made by the searches with the defined SCOP domains and used the structural information already available for 398 DUF families to evaluate the success, precision and error rates.

The success rates range from 60 to 94 % with the highest rate reported by HHsearch method (Additional file 1: Table S1). Fold assignments by searches in SCOP-NrichD and SUPERFAMILY database also showed very high rate of correct fold assignments, of 92 and 93 % respectively. Lowest success rate was observed for predictions by pDomTHREADER. For 16 DUFS predicted by pDomTHREADER, SCOP definitions were unavailable at the time of analysis. The availability of these domain definitions, in future, may improve the observed success rate. Lowering of the P-value cut-offs for pDOMTHREADER, to include more families, however, resulted in a concomitant increase in the error rate by 6 %.

With respect to wrong assignments of folds, very low error rates (below 1 %) were observed for all the five methods. No false positives were detected in searches with HHsearch and SCOP-NrichD database and very few incorrect assignments were made using SUPFAM+ database, pDomTHREADER and SUPERFAMILY (2, 1 and 1 in number respectively). These general trends for the 398 proteins of already known structure affirm that the parameters used for each method in this study were stringent, resulting in hits with high confidence (see Additional file 2: Table S2 in the context of assignment of folds to DUFs).

### Performance of various methods employed in the study

**Fig. 1:**
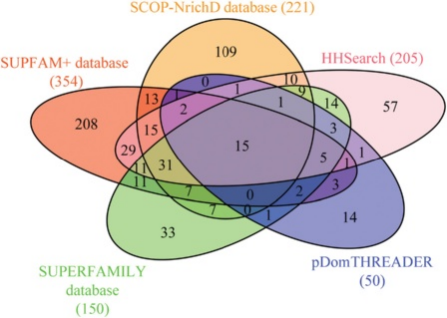
SCOP-superfamily assignments by each method: Venn diagram illustrating the number of remote similarity detections that are unique and common to the five methods in the study

The computational design of artificial ‘linker sequences’ is an effective way of filling voids in protein sequence space [22, 23]. We had already demonstrated that such ‘artificially designed sequences’ can improve the sensitivity of commonly employed search methods in the detection of distant protein similarities. That the databases enriched with designed linkers are amenable to generic searches was shown with 417 novel connections between members of various Pfam families [22]. In the present assessment, we have attempted to associate the domains of unknown function to a known fold through five different and powerful computational structural genomics approaches, including the SCOP-NrichD database searches with the hope that clues to a potential function can be identified. As seen in Fig. 1, in terms of number of connections, both the SCOP-NrichD and SUPFAM+ database identify high number of hits (109 and 208 unique hits respectively). Fifty-seven and 33 unique hits are identified by HHsearch and SUPERFAMILY database searches alone. Fourteen unique hits are identified by pDomTHREADER. In the following sections, we discuss the results obtained from each of the five methods/ searches employed in the study and subsequently highlight examples where more detailed annotation provides important clues to improve the functional annotation of the DUF families. Several of the SUPFAM+ connections identified were through a member of known structure in one of the grouped Pfam families in SUPFAM+, akin to Pfam Clan-based association.

We have, therefore, chosen to highlight examples from the SCOP NrichD database searches since these are more direct one-to-one cases of association between the DUF family and the SCOP domain and also have been applied for the first time.

### Structural annotations by each method

In the present study, 3,087 DUF families with unknown structure and function were chosen as targets and form the query dataset. HMM profiles or representative sequences from these families were queried against sequence or profile databases with structural information using available remote similarity detection methods. The results from these individual methods were then combined together to reduce ambiguity and improve confidence in assignment.

#### SCOP-NrichD database

SCOP-NrichD database consists of natural sequences from SCOP database and sequences of their homologues, which are enriched with computationally designed intermediate sequences. These designed sequences between related protein families when augmented into natural sequence databases, act as linkers and show remarkable enhancements in detecting remote homologues. The strength of such enriched database lies in their ability to connect related proteins families in the sequence space and facilitate in detecting non-obvious connections [22]. The SCOP-NrichD sequence database is amenable to both sequence-based and profile-based searches. We used both ‘jackhmmer’ as well as ‘hmmsearch’ [28] to search in the SCOP-NrichD database. To achieve maximum coverage, searches were also made in natural sequence database (SCOP-DB) [23]. This study presents the first large-scale implementation of remote-similarity detection using artificially enriched sequence database and provides SCOP superfamily assignments for 245 of the 3,087 DUFs in the query set. Amongst these, predictions for 24 DUFs were discarded, as the SCOP superfamily predicted by the other methods were dissimilar. Thus, domain assignments by searches in SCOP-NrichD database could be assigned to 221 DUF families. Interestingly in certain cases such as DUF455, DUF1495 and DUF2551, only designed intermediate sequences could be detected in homology searches and since these designed sequences were previously annotated with the SCOP family information between which these were designed, we were thus able to relate them to possible folds.

#### SUPFAM+ database

SUPFAM+ database is a comprehensive database of non-trivial evolutionary relationships between functional families derived using their structural information [24]. Briefly, the method employed in the SUPFAM+ database first relates Pfam families (with or without known structures) to SCOP superfamilies using a rigorous profile-profile alignment method, AlignHUSH [29]. Secondly, it identifies relationships amongst Pfam families and then combines all the identified relationships to derive a mapping between Pfam families and SCOP structural families (direct or indirect). Using the information provided in the database, evolutionary relationships were derived for 384 of the 3,087 DUF families, of which 30 were removed from further consideration, owing to differences in results between the methods. Amongst the remaining 354 cases of distant similarity detection, 312 families could directly be related to SCOP domains, whereas the remaining 42 DUFs were indirectly related to a structural superfamily.

#### SUPERFAMILY database

One of the most widely used domain assignment methods to identify structural domains in newly sequenced proteins is by searching against the SUPERFAMILY database. This database provides 15,438 HMMs representing 2,019 SCOP superfamilies, which can be searched using the ‘hmmscan’ program from HMMER3 [28]. Using this approach, remote relationships could be identified for 173 DUFs. However, 23 of these predictions were excluded due to dissimilar assignments across the methods. Therefore, predictions for 150 DUF families were finally obtained using superfamily domain assignments.

#### pDomTHREADER

Protein threading is an approach to detect the protein fold for a sequence using a detailed representation of the known 3-D structures. The method involves threading of the query protein sequence onto known structures and their fitness is calculated using knowledge-based pair potentials. One such widely used algorithm is pDomTHREADER, which also provides domain boundaries [26]. Using this method, structural annotations were made for 68 DUF families. Predictions were made for 13 more DUF families, however since mapping of the template PDBs to their cognate SCOP fold was not available, these were excluded from our study. Out of these 68 predictions, 18 superfamily assignments were excluded as they resulted in dissimilar annotations, leaving predictions for 50 DUF families for further analysis.

#### HHsearch

HHsearch is one of the best performing software suites for detecting remote protein relationships and generating accurate alignments for homology modelling [19]. HHsearch queries a multiple sequence alignment or HMM against a library of HMMs (see Methods for details). For 205 DUFs, remote relationships could be detected using HHsearch, excluding out predictions for 22 DUF families with dissimilar annotations.

In all, 614 DUFs could be associated with a structural fold through the five methods employed here (Additional file 3: Table S3). Individual predictions and results files for each of the 614 DUFs can be accessed through http://proline.biochem.iisc.ernet.in/RHD_DUFS/. This web-resource provides an interactive and searchable table with hyperlinked DUF families and their SCOP-superfamily predictions to their respective Pfam and SCOP database entries. The table also provides links to result files of individual methods, wherever available. These results file are parsed at the employed search criteria and also provide sequence alignment between the query sequence/profile and the domain hits. We have also made available online, the result files for the remaining 2,473 DUF families that were associated with a structural fold at low confidence http://proline.biochem.iisc.ernet.in/RHD_DUFS/ALL_RESULTS. Since this was a large-scale assessment, E-value and coverage filters were applied to report true and high confidence hits. Therefore, the set of poor confidence hits reported here, qualified the E-value cut-off employed in the search method but failed the imposed coverage criteria. The users may employ their discretion to evaluate these results.

### Structure and functional annotation for uncharacterized protein families using multiple methods

The rationale for using different remote similarity search methods is to maximize coverage and to provide confidence to each homology detection. As evident here, the results would be less impactful if homology detection from individual methods only were considered (see Fig. 1). Upon combining the results from all 5 methods and removing 46 ambiguous predictions, 614 DUFs could be related to SCOP superfamilies. These 614 families encompass about 22 % of the uncharted sequence space with nearly 237,802 sequences. Since the results show consensus, independent of the method employed, they may be considered as reliable detections of relatedness. Amongst these 614 annotated DUF families, 15 and 39 are “high” confidence results since they are recognized by all five and four of the methods respectively. Available Pfam clan information for 46 of these high confidence cases also supports these results (Additional file 3: Table S3). Indeed, detection of distant relationships made here is in agreement for nearly 100 % of the DUF domains with already available clan information. Relationships involving 50 and 89 DUF families, recognized by three and two methods respectively, are referred to as “medium” confidence hits and the remaining relationships involving 421 DUFs recognized by a single method are considered as “low” confidence. A Venn diagram of ‘overlaps’ between the five methods clearly illustrates that the capabilities of each method to detect remote relations are different and often non-overlapping (Fig. 1). Further, as shown in Fig. 1, results from HHsearch method and SUPFAM+ database show a substantial overlap (109 cases), which may be attributed to both methods employing a profile-profile alignment based search. We also observe a marked overlap between HHsearch and domain assignments using SUPERFAMILY database (89 cases). Similarity amongst these methods may arise due to similarity in the query database of HMM models for all SCOP families. A significant proportion (68 %) of the relationships is obtained by a single method. A large fraction of these results are from searches in SUPFAM+ database, followed by SCOP-NrichD database. Although the success rate for SUPFAM+ database method was found to be relatively lower than other methods (Additional file 1: Table S1), indirect mappings aid in detecting many such relationships (see Methods for details).

SCOP-NrichD database strategically fills-in the gaps between related families and helps in detecting 109 unique, previously undetected remote relationships [22]. Recognition of relationships involving DUF1765, DUF2683, DUF2889 and DUF3489 families, with SCOP superfamilies was made only in searches in SCOP-NrichD database, employing designed-intermediate sequences. These designed intermediate sequences annotated with the parent SCOP families information, enabled us to associate a possible structural fold. Such a case was also observed during assessments of methods wherein relationship between DUF4352 and Immunoglobulin fold was recognised using designed sequences. The single known structure for this family also conforms to an Immunoglobulin fold thereby confirming that such intermediate sequence can lead to correct fold recognition.

423 DUF families involved in the recognition of distant relatives did not contain any Pfam clan information. 343 of the DUF families involved in detected relationships were made exclusively through any one of the methods employed here with both SCOP-NrichD and SUPFAM+ accounting for 105 and 165 relationships respectively. Recognition of relationship using HHsearch could be made for only 34 families. Superfamily and pDomThreader aided detection of relationships for 26 and 13 families respectively (Additional file 4: Table S4 and Additional file 5: Figure S1).

### Phylogenetic diversity of the DUFs

**Fig. 2:**
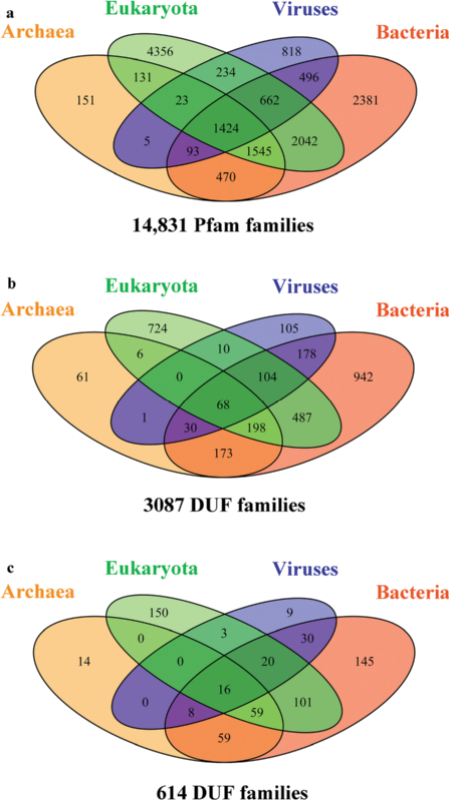
Venn diagrams representing distribution of families in different biological kingdoms. **a** The distribution of all Pfam families **b** the distribution of 3,087 DUFs with no structural or functional information and **c** the distribution of 614 DUFs with SCOP superfamily assignments, in different kingdoms

Here we examine the distribution of 14,831 Pfam families, 3,087 DUFs and that of 614 DUF families in different phylogenetic kingdoms. Compared to 10 % of the Pfam families that are found in all major kingdoms of life *i.e.* archaea, bacteria, viruses and eukaryotes (Fig. 2a), only a small proportion (68 families out of 14,831 Pfam families) of these have no known structure or known function (Fig. 2b). A significant proportion (30 %) of DUFs is found only in bacteria. 23 % of DUF families are restricted to eukaryotes and about 16 % belong to both bacterial and eukaryotic species (Fig. 2b). This uneven distribution of DUFs suggests that these families are more likely to be conserved in a specific organism or for specific environmental conditions and unlikely to be a part of housekeeping or constitutive proteins [4, 30]. Figure 2(c) shows that relationships detected for 614 DUFs encompasses structural annotations for higher organisms, wherein 150 families belong to eukaryotes. Almost equal number of families are annotated for bacterial species (145 families), followed by 101 families found in both bacteria and eukaryotes. Although only 68 families found in all kingdoms have no structural and functional information, we provide structural annotations for 16 such families.

### Distribution of folds and superfamily

**Fig. 3:**
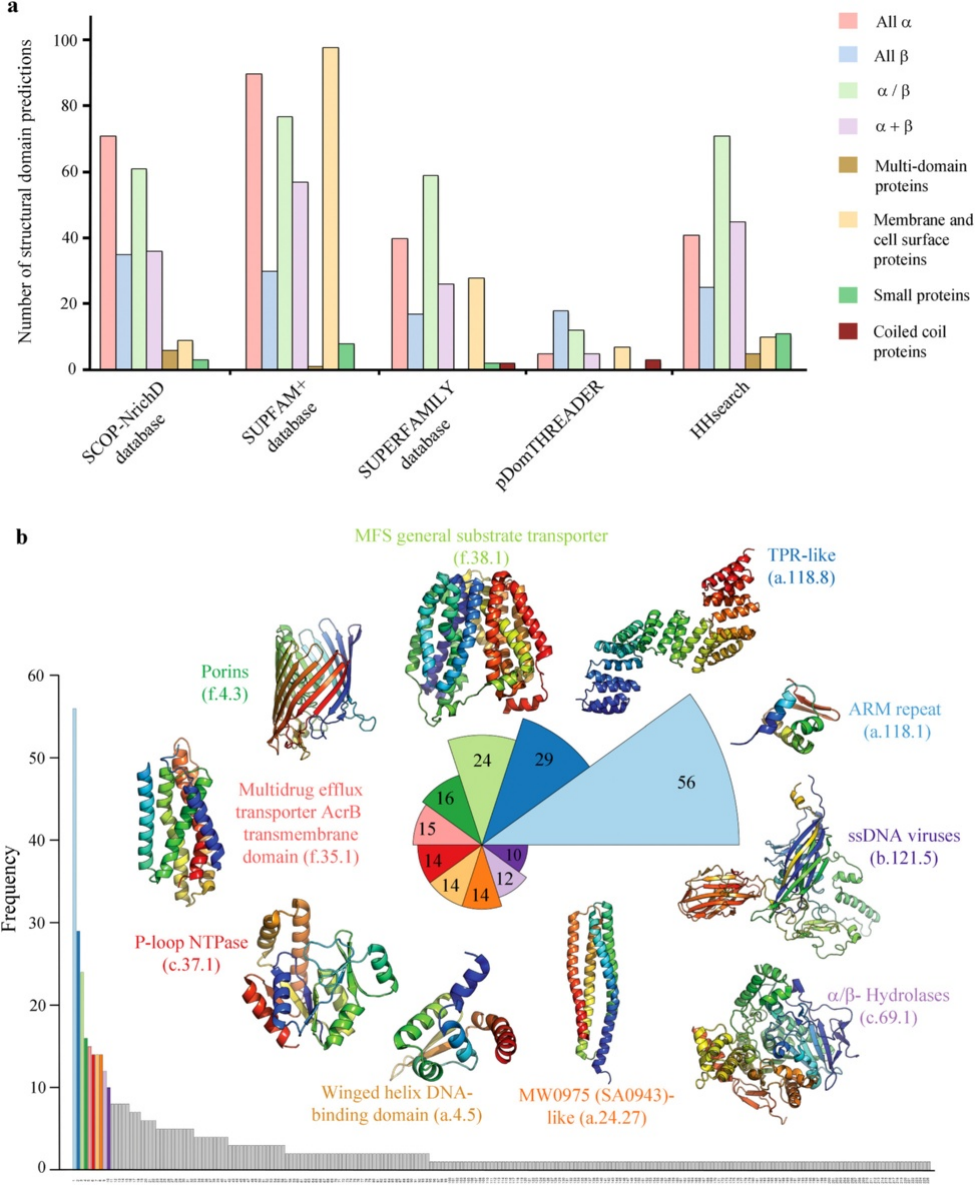
Distribution of combined remote similarity detection across different SCOP classes: **a** SCOP Class distribution of 628 domains recognized as related to 614 DUF families for each prediction method. **b** Bar plot representation of the frequency distribution of all SCOP superfamilies represented in structural domain recognition. Representative structures of top 10 superfamilies are shown around the radial frequency plot of these superfamilies

The SCOP superfamily recognition for 614 DUFs reveal 628 SCOP domains, which belong to 226 different SCOP superfamilies and 173 different SCOP folds. In our study, almost 70 % of the structural annotations are from the three major SCOP classes: the all α class, α/β class and membrane proteins. Figure 3(a) shows the distribution of 628 distant similarity detections across major SCOP classes for each of the 5 methods and Fig. 3(b) depicts the frequency distribution of the top-10 SCOP superfamilies involved. ARM repeat, TPR-like, MFS general substrate transporters, Outer-membrane protein A-like and Multidrug efflux transporter AcrB transmembrane are among the most frequent superfamilies involved in distant relationships. It is noteworthy that not all methods contribute to similar fold recognition. For instance, SCOP-NrichD database aids in relationship detection in protein folds with more than two families, for which intermediate sequences could be designed. Success of searches in SCOP-NrichD database is therefore limited in orphan folds (folds with only one family) such as Multidrug efflux transporter AcrB transmembrane domain and MFS general substrate transporter fold. For folds such as α-α superhelix many intermediate sequences (331,042 designed sequences) could be designed which indeed have proven to be helpful in detecting remote relationships. Other methods such as SUPFAM+ database, SUPERFAMILY database and HHsearch rely only on diverse sequences representing a protein fold. Many of these domains of unknown function are found to be essential DUFs based on the presence of essential proteins in these families. Goodacre and co-workers report 238 DUF families as essential DUF families in bacterial species [4]. We observe that 33 out of 614 DUFs form essential families (see Additional file 3: Table S3).

### Highlights

The computational structural genomics approach to DUF family annotation adopted here is an attempt to extract function information for poorly characterized sequences that show low sequence similarity to well characterized protein families. This is done through the assignment of the closest structural domain using sensitive profile-based approaches. The use of structural information in either the method or the dataset adopted here, it is anticipated, will provide the right pointers with high confidence especially when they all offer the same solution.

Confidence in reporting hits was associated with the number of methods that could associate a DUF family to a fold in SCOP. As shown in Fig. 1, each method varied in the number of unique hits. Further, the most common fold associations observed for the DUF families were repetitive domains such as the ARM repeat and TPR. While in these cases, the association is useful in appreciation of the potential structure that might be adopted by the proteins, it is difficult to associate their biological and functional roles. We therefore examined the hits that were associated with a potential enzymatic role (through enzyme association of the cognate structural member identified in the searches) (Additional file 3: Table S3 and Additional file 6: Table S5). Further, we also considered results from searches made in the SCOP-NrichD database since these were a) direct associations to the SCOP fold involving natural or artificial linkers b) ranked high in the number of unique hits identified in the five different methods. For each of the families we also obtained the similarity detection for a representative member using MESSA [31], a meta-server that integrates structural and functional predictions using select tools and additionally, also submitted the seed sequences from each of the DUF families to the CD search tool to detect conserved domains [32].

### DUF3050 (PF11251)

**Fig. 4:**
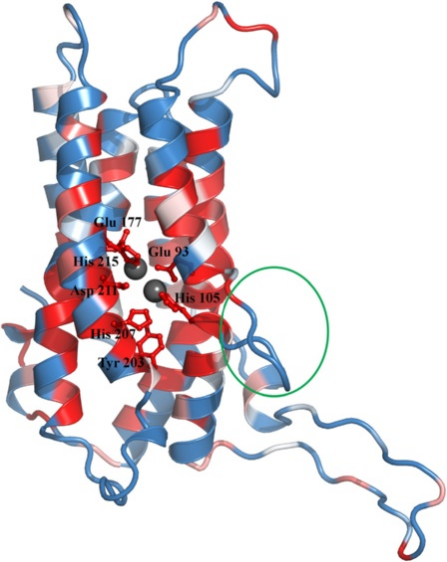
Modeled structure of DUF3050 using 1RCW as template. The cartoon representation of the structure is coloured based on sequence conservation (from blue to white to red, where blue indicates poorly conserved residues and red indicates highly conserved residues). The metal-coordinating active site residues in the di-iron sites, Glu 93, His 105, Glu 177, His 207, Asp 211, and His 215 and other residue in the active site (Tyr 203) are depicted in ball-and-stick format

DUF3050 consists of single domain proteins of roughly 230 residues from nearly 250 bacterial species. Four methods employed here relate this domain to members of the Heme-oxygenase-like superfamily, consisting of many redox enzymes. The fifth method, pDomTHREADER also points to Iron-containing redox enzymes. Additional support is provided from Pfam Clan database (CL0230: Heme oxygenase) and searches in the MESSA server as well. Conserved domain searches for seed sequences of DUF3050 however, do not find any hits. Alignments in the SCOP-NrichD database show that this domain family aligns with CADD-like proteins (Chlamydia protein associating with death domains), which has been shown to induce apoptosis when transfected into mammalian cells [33]. We generated an alignment for the DUF3050 family and modelled a representative member using CT610 from *Chlamydia trachomatis* (PDB: 1RCW [34]) as template, to obtain a seven-helix bundle with a potential di-iron center, likely forming the active site (Fig. 4). In the template, Fe1 in the active site is coordinated with a glutamate residue (Glu 93), two histidines (His 105 and His 207) and a water molecule. Fe2 is coordinated by histidine (His 215), two glutamates (Glu 177, Glu93), aspartate (Asp 211) and a bridging water molecule. These residues are found to be highly conserved in the DUF3050 family (Fig. 4). Tyr170, in close proximity to the di-iron center and potentially forming a tyrosyl radical, is also conserved in the DUF family. An insertion is observed in the DUF3050 family in a region involved in the dimer formation in the template (encircled in green in Fig. 4). These studies show that this domain family likely functions as an oxidoreductase. Its similarity to the CADD proteins, which were hitherto considered unique to the Chlamydia species, needs to be further investigated by assaying for binding with the DR5 (death receptor) domain for a potential role as a toxin that can induce apoptosis.

### DUF 1572 is related to DinB/YfiT-like putative metalloenzymes

**Fig. 5:**
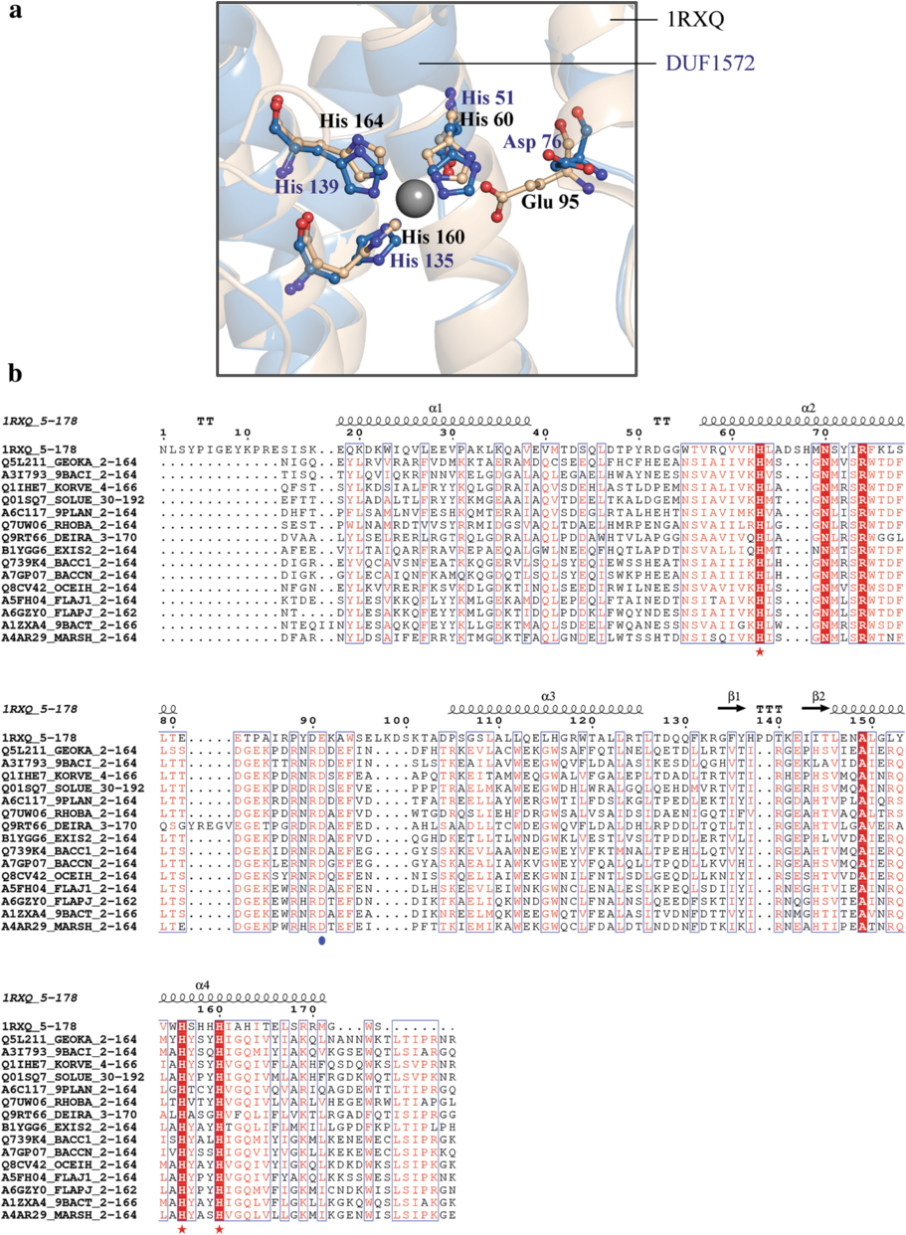
DUF1572 – a putative metalloenzyme: **a** Structural alignment of YfiT from *Bacillus subtilis* (PDB ID: 1RXQ, shown in wheat color) and modeled DUF1572 (light blue) highlighting the active site region. Conserved histidine residues coordinating with the Zn metal ion in both structures are shown in ball-and-stick. **b** Multiple sequence alignment of representative sequences of DUF1572 with the sequence of 1RXQ, highlighting the conserved histidine residues by red stars. A blue circle denotes the additionally conserved active site Aspartate residue

DUF1572, encompassing several hypothetical proteins in bacterial species such as *Bacillus, Thermaerobacter* and *Sporosarcina,* finds hits to DinB/YfiT-like putative metalloenzymes with very high confidence (*i.e.*, by all 5 methods). DUF1572 is a member of Pfam Clan - DinB (CL0310) that includes DUF1569, DUF1706, DUF1993 and DUF664 [5]. Metals are predominantly involved in functions related to electron transfer, and are found as cofactors in enzymes serving as electrophiles or nucleophiles. Sequence alignment and structural superimposition of a modelled structure of DUF1572 (UniProt: Q5L211_GEOKA) with YfiT from *Bacillus subtilis* (PDB ID: 1RXQ [35]) as template, reveals the conservation of histidine residues that can potentially coordinate a metal-ion (Fig. 5a). Despite very low sequence similarity, the four-helix bundle structure and the conservation of three histidine residues in DUF1572 and additionally conserved residues in the active site are indicators of metal-dependent hydrolase function (Fig. 5a and b). The conservative substitution of glutamate residue (Glu 95), involved in the binding site, with an aspartate residue in the DUF family, may affect the binding affinity or rate of the reaction.

### Enormous divergence and mutation of functional residues during evolution

The classification of proteins into functional and structural domain families relies on the conservation of sequence, structural and functional signatures. A point we would like to make here is that in several instances, commonly applied search tools, integrated servers and the methods adopted here converge appreciably and point the DUF families to a protein family of known function. This is a consequence of improved strengths in search procedures and the quality of annotation of various datasets. However, on close examination of the alignments with the associated parent family of known function, we found that although a majority of the structural and functional signatures are conserved, some of the critical residues that dictate shape (or fold) or function (catalytic residues) are missing. Examples of these families in our detailed annotation efforts are many, as in the case of DUF 2071, DUF 1636, DUF2092, and others. Clues on potential structure and function are also obtained through clan membership, where any one member may have a structure or function characterized. However, here also, on examining the binding site residues, the hallmarks of the family with which associated, are missing. Therefore, in such cases, the predictive power of such integrated approaches will be limited to offering useful pointers on the potential fold. Where such critical residues are missing, we can only speculate that such proteins, like pseudo-kinases/kinase-like proteins, are either artifacts of a functional member or demonstrate a hitherto unknown specificity for an altered substrate [36]. Possibly, these could also be examples of the re-invention or the primordial instances of the utilization of these folds for a hitherto undiscovered function or substrate, as the case may be.

We discuss three examples of domains where functional signatures are only partially conserved and show that function annotation is ‘non-trivial’ even when all methods concur on detection of distant relationships.

### DUF2071 is related to ADC-like fold

**Fig. 6:**
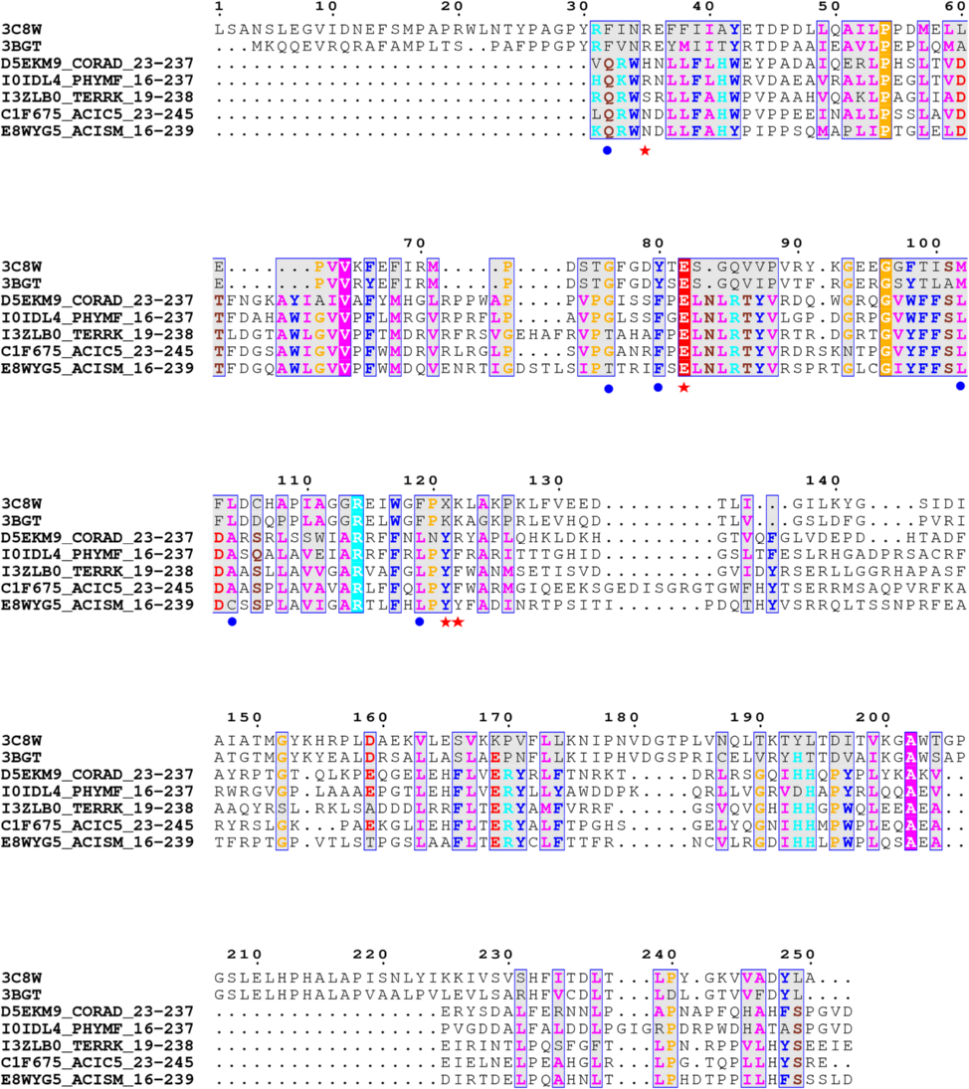
A multiple sequence alignment of the DUF2071 family with acetoacetate decarboxylase. Alignment of representative members of the family with the structural templates (3BGT:A, 3C8W:A). Hydrophobic and active site residues are shown (blue circle, red star respectively)

PF09844 is an uncharacterized COG protein domain family (DUF2071) that occurs in many bacteria and archaea. It is known to be similar to ygjF that occurs in many prokaryotes (mismatch-specific uracil DNA glycosylase). Three of the five methods employed here namely searches in SCOP-NrichD, Superfamily and HHsearch relate it to the ADC-like fold (Acetoacetate decarboxylase fold). This association is also supported by Pfam clan, which groups this domain into the ADC-like clan, CDD searches and reports from the MESSA server. The link to this fold member in SCOP-NrichD searches is mediated through 3C8W (a potential acetoacetate decarboxylase (ADC) (Q5ZXQ9) from *Legionella pneumophila subsp. Pneumophila*, at an E-value of 3.1e-62 and with 38 % identity. The ADC catalyses the conversion of acetoacetate to acetone, a key component in the anaerobic metabolism of carbohydrates in some bacteria. Studies have shown that the reaction of ADC proceeds through a Schiff-base intermediate formed by the reaction of Lys 115 with substrate [37]. The cone-like arrangement of the structure harbours hydrophobic residues in its hollow core that stabilize the active site Lys 115. Although not directly involved in any side-chain interactions, Lys 115 projects into the core presumably to anchor the substrate through a Schiff-base intermediate and to orient it into the channel such that it may be acted upon by polar residues deeper into the core. Substitution mutations of the lysine 115 have shown to render the enzyme inactive [38] and therefore critical. But for the active site residue, residues in the binding pocket such as Glu 76 is conserved in all the members (Fig. 6). Hydrophobic residues such as Phe 26, Gly 71, Tyr 74, Met 96, Leu 98 and Tyr 113 in the binding pocket are well conserved/conservatively substituted. The crucial Schiff-base forming Lys 115, however is not conserved in PF09844. Although all pointers report similarity with this fold, the functional site residues are not entirely conserved.

### DUF 2092 is related to prokaryotic lipoproteins and lipoprotein localization factors

**Fig. 7:**
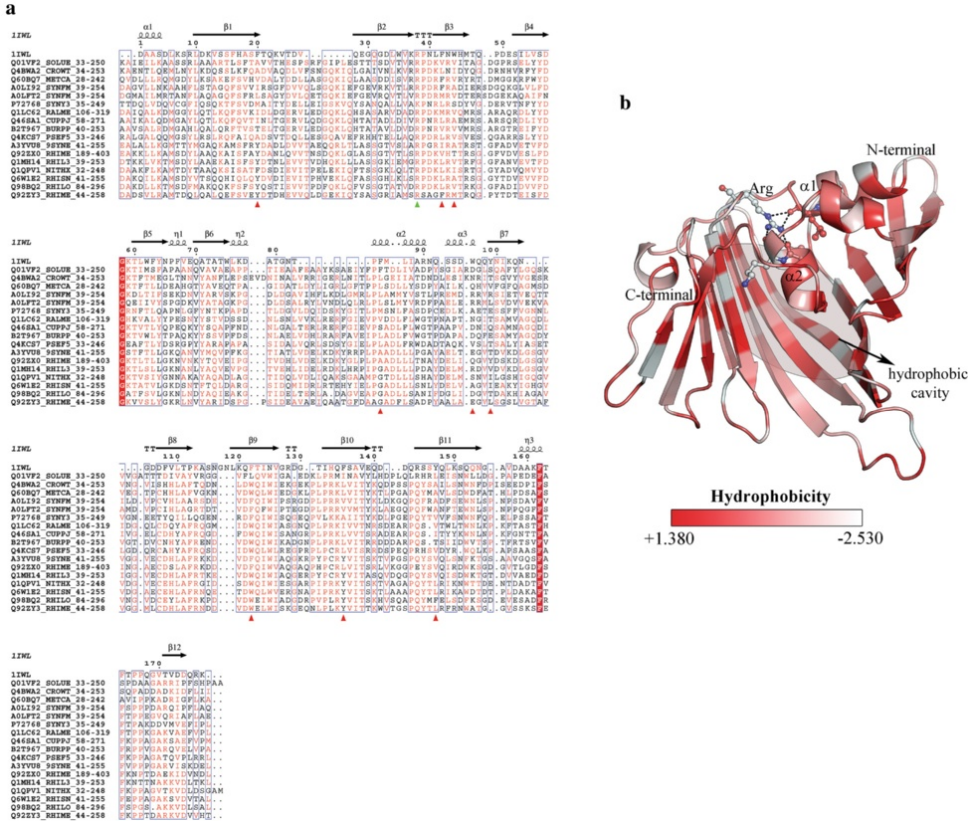
DUF2092 – a lipoprotein localization factor, LolA: **a** Multiple sequence alignment of representative sequences of DUF2092 and bacterial lipoprotein localization factor. Residues involved in the hydrophobic cavity shown with red triangles. **b** Modelled structure of a DUF2092 with bacterial lipoprotein localization factor, LolA (PDB ID: 1IWL) as template depicting the prokaryotic lipoprotein and lipoprotein localization factor superfamily. Residues are coloured based on a hydrophobic scale ranging 1.380 to −2.530 denoting the most hydrophobic to least derived from Eisenberg normalized hydrophobicity scale [59]

DUF2092, a conserved hypothetical domain of 215 amino acids and four different domain architectures has been associated with the Outer-membrane lipoproteins carrier protein (LolA) with very high confidence. Five highly conserved Lol proteins are involved in the sorting and localization of lipoproteins [39]. The structure of LolA enfolds a hydrophobic cavity consisting of an unclosed β-barrel and a α-helical lid. The cavity represents a possible binding site for the lipid moiety of lipoproteins and is partly conserved in this family of proteins. In Fig. 7(a), residues constituting the hydrophobic cavity are highlighted in the multiple sequence alignment of selective members of DUF2092 family. All positions are not highly conserved; however the hydrophobic nature of the binding site is conserved. Additionally, a conserved arginine residue involved in the opening and closing of the localization factor (LolA) is seen in the DUF2092 family and indicated with a green triangle (Fig. 7a and b).

### DUF1636 is related to Thioredoxin-like [2Fe-2S] ferredoxin family

**Fig. 8:**
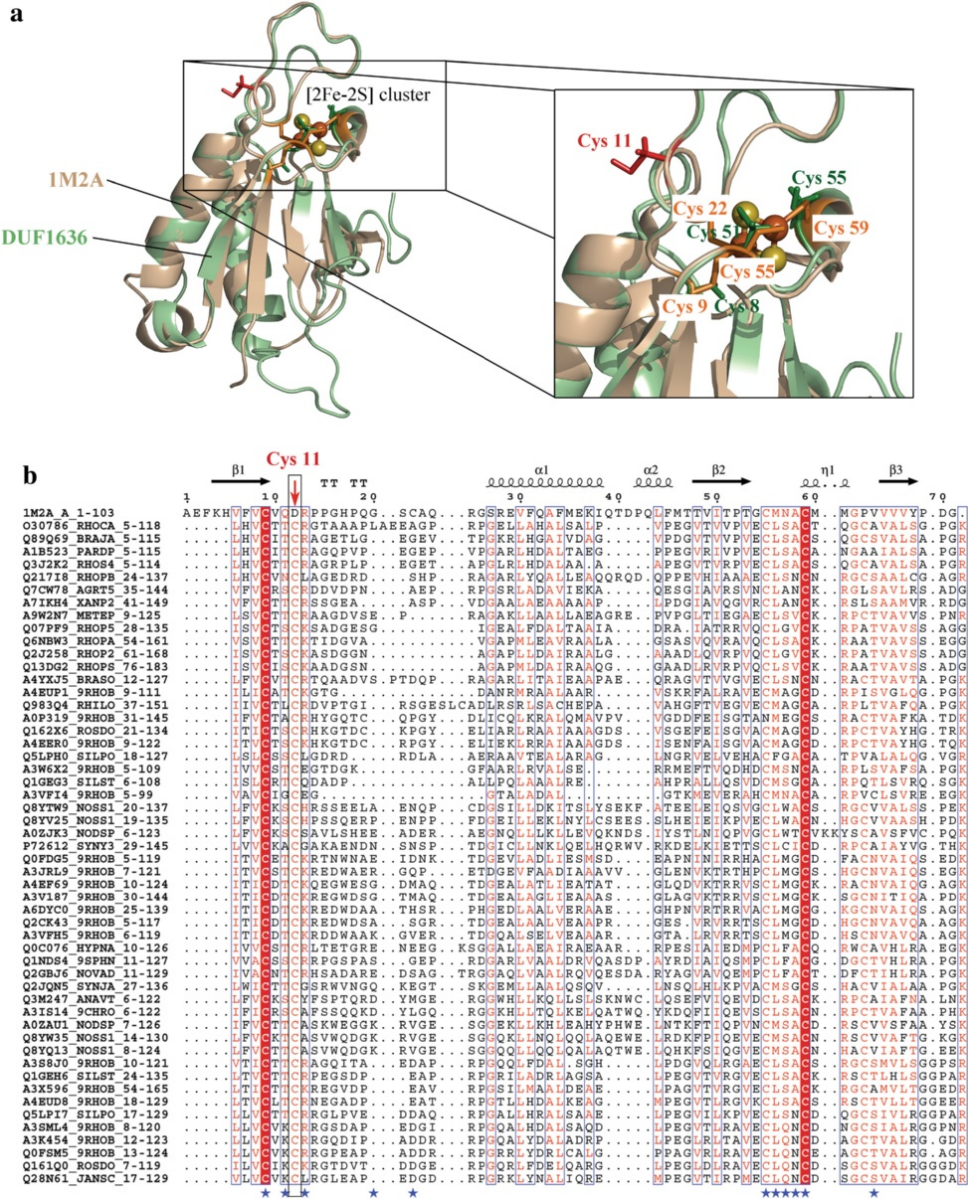
DUF1636 – a Thioredoxin-like fold: **a** Structural alignment of crystal structure of wild-type thioredoxin-like [2Fe-2S] ferredoxin from *Aquifex aeolicus* (PDB ID: 1M2A, shown in wheat colour) and modelled DUF1636 (green colour). The conserved cysteine residues in the active site and in the loop region are shown in ball-and-stick. **b** Multiple sequence alignment of representative sequences of DUF1636 and 1M2A with active site residues marked with blue stars. A red arrow highlights the conserved cysteine residue in the loop region. For clarity, only first 70 residues containing the active site are shown

DUF1636 (130 residues) encompasses several single domain hypothetical proteins of prokaryotic origin that are connected to the Thioredoxin fold (Thioredoxin-like [2Fe-2S] ferredoxin family). Figure 8(a), shows the characteristic thioredoxin-fold of the model of a representative member (UniProt: O30786_RHOCA) derived using 1M2A as the crystal structure template (Thioredoxin-like [2Fe-2S] ferredoxin from *Aquifex aeolicus*). Proteins containing [2Fe-2S] clusters are known to participate in many important biological processes associated with oxidation-reduction reactions. The [2Fe-2S] cluster in the Thioredoxin fold is located near the surface of the protein and the four cysteine residues interacting with the [2Fe-2S] clusters are located near the ends of two surface loops. Mutational studies of cysteine residues reveal that Cys 9 and Cys 55 in 1M2A are found in the interior of the protein and therefore more rigid and highly conserved, while other two are more amenable to mutations [40, 41]. Sequence alignments of DUF1636 show the conservation of three of the four cysteine residues binding to iron-sulphur cluster and an additional atypical cysteine residue (Cys 11) which possibly compensates for the non-conserved Cys residue at position 24 (Fig. 8b). Our binding site analysis and derived model shows that the members of this family are likely involved in cellular redox homeostasis.

### Caveats in recognizing functions from sequences and structures

The complex relationship between protein structure and function presents a major challenge in gaining inferences on functions from the structure. The problem is compounded by versatile folds that provide a robust scaffold for different functions such as the TIM β/α-barrel, Rossmann, ferrodoxin, P-loop NTP hydrolase, and Thioredoxin-like [42–44] folds. Enolases, N-Acetylneuraminate Lyase Superfamily, and Cronotase superfamily are classic examples of highly diverged enzyme superfamilies whose members catalyze different reactions [45–47]. On the contrary, there are several evidences of enzymes catalyzing similar reactions with no clear sequence or structural similarity. Glycosyl hydrolases (EC number 3.2.1) are associated with more than 100 different families [48]. Likewise Acid phosphatase (EC 3.1.3.2) function is found to be associated with more than five different folds [49]. The concept of common ancestor or homology although useful, however may not always imply same function. Function recognition is further complicated by protein-protein interactions, multimeric functional states, paralogous sequences that often do not conserve function, or the multifunctional nature of few proteins [50–52]. As a consequence, function assignment through homology may lead to erroneous or incomplete annotations and therefore, requires immense caution for structure-based functional assignment. Additional information such as sequence signatures, gene neighbourhood, domain architectures, interacting partners, conserved catalytic triads or motifs, functionally conserved active site residues, organism specificity, co-variation across metagenomes and interacting ligands can add confidence to function predictions [50, 53].

Attempts on recognition of functions of protein domains even with a known structure are not always successful. For example, members of DUF1048 have left-handed superhelix-fold, however no data is currently available to describe the function. In our study, evolutionary relationships for members of DUF1637 suggest a cupin-like fold. However the critical functional residues (Cys93 and Tyr157) in cysteine dioxygenase function are found to be mutated in this DUF family, where the cysteine residue is replaced by a conserved, small, hydrophobic residue and no conservation is observed at the Tyr 157 position. Similarly, our predictions for DUF354 family suggest its function as Glycosyl transferase. However, upon detailed analysis of the catalytic residues, very few were found to be conserved in this DUF family and thus, its function still remains obscure.

Nevertheless even with these limitations and the complex inter-relationship between structure and function, structural information can help focus on the functional space [30]. GO terms or enzyme classifications for these folds may be helpful in guiding further experiments. Protein-level function annotations are intensively curated, however, often ignoring the context of structural domains. Considering the hierarchical organization of GO as well as structural domain classification, DcGO database provides a unique resource for structural domain-centric Gene ontology annotations [54]. Using these GO annotations for SCOP superfamilies and families, we also provide a detailed list of GO annotation for each predicted SCOP superfamily for 614 DUFs (Additional file 3: Table S3). Additionally, using the EC number to PDB mapping for single domain enzymes provided by RCSB Protein Data Bank (see Methods for details), we also provide a list of possible enzyme reactions for each SCOP superfamily (Additional file 3: Table S3).

### Ambiguous predictions

Although the results reported in this study exclude the 46 ambiguous cases (see details in Methods), they enable us to make some interesting observations. On careful analysis, it was found that predictions for 17 DUF families belonged to the same SCOP fold, providing a plausible clue about the topology and architecture of the family members (Additional file 7: Table S6). Amongst the remaining 29 DUF families, where predictions belong to different SCOP folds, some included examples such as 6-bladed β-propeller, 7-bladed β-propeller and 8-bladed β-propeller which are believed to be evolutionarily related [55]. Although these cases are regarded as ambiguous or low-confidence cases, they may show evidences of the continuous nature of the protein fold space. Significant sequence conservation or local structural resemblance and functional similarity can indicate evolutionary relationships between proteins despite noticeable structural differences at the fold level [56, 57].

### Short length of domains, repeats and domain definitions can limit the scope of function recognition

An underlying assumption in such annotation efforts is the globular nature of the proteins, however, if they constitute non-globular segments such as coiled-coils, low complexity regions, transmembrane regions or long loops, then the annotation of such cases based on known protein domain families is even more challenging [58–60]. A large number of domains are short with less than 100 residues. As seen in (Additional file 6: Table S5), for 39 domains with potential enzymatic role, short length of the query domain and alignments limited the scope of function recognition. Many alignments did not involve active site residues or residues playing other structural and functional roles. DUF4070, is a domain that is found at the C-terminal end of Radical Sam enzymes. The N-terminus of Radical SAM enzymes binds the substrate and contains a 4Fe-4S cluster and two SAM binding domains. The C-terminus is believed to likely involve in shielding the substrate from the solvent [61]. Similarly members of DUF1298 family are found to occur at the C-terminus of O-acyltransferase WSD1 (approximately 170 residues), however, no functional role has been assigned to this domain so far.

DUF659 family comprises of Transposase-like protein with no known function. This family consists of almost 65 different domain architectures predominantly consisting of Zinc-binding domain at the N-terminal and/or a C-terminal dimerization region. Queries from DUF659 family detected members of the Ribonuclease H-like superfamily with high confidence (4 out of 5 methods). The alignment between DUF659 and RNase H-like proteins showed very high query coverage (~97 %), however the coverage for the hit was observed to be less than 50 %. On detailed examination of the alignments, it was deduced that DUF659 is a part of the fully functional RNase H-like family [62] and therefore, no functional inferences could be derived. Interestingly, the full length sequences containing the DUF domain when searched in SCOP-NrichD database and SUPERFAMILY database, showed the presence of RNase H-like overlapping with the DUF domain (Additional file 8: Figure S2) at statistically significant E-values.

## Conclusions

Concerted efforts including protein crystallography, co-expression studies, protein interaction assays and functional assays are required for reliable structural and functional annotations for conserved protein families. However, these methods can be time and resource consuming. While earlier, large-scale efforts for recognition of structural or functional information for DUF families relied greatly on a single but sensitive homology detection method [63, 64], here we present a ‘computational structural genomics’ approach, by using various remote similarity detection methods with unique strengths to annotate families with unknown functions. Artificially enriched sequence databases, which have previously shown promising results in recognizing relationships between highly diverged protein families, have been used for the first time to establish remote relationships on a large scale. Other advanced methods such as sequence-to-profile searches against HMM library (SUPERFAMILY database and HHsearch), fold recognition by pDomTHREADER and SUPFAM+ database are also employed to achieve better coverage and reliability of hits. To this end, we provide structural clues for 614 DUFs and additional functional clues by virtue of associated GO terms and enzyme reactions, wherever possible.

Predominantly predictions suggest that many families may be involved in membrane transport or function as transcription regulators. Many have been reported as essential families in bacterial species, which can be further explored experimentally. This approach when used in conjunction with detailed structural analysis can lead to the identification of critical functional residues in the protein of interest. This has been elucidated by the case studies used; wherein high levels of granularity have been achieved. Individual amino acids contributing to protein function have been identified. This pipeline can be used to not only annotate DUFs, but can also be used as a tool to perform in-depth analysis.

## Methods

### Families with unknown structure and function

Domains named ‘DUF’ or ‘UPF’ or description containing ‘unknown function’ or ‘Uncharacterized protein family’ were identified from Pfam database (version 27.0) [62] and HMMs for these 3,786 DUF and UPF families were retrieved from the database. Amongst these, 699 DUF families have solved structures of which for 398 DUFs, SCOP domain annotations are also known; these were used as test dataset. Thus profiles of remaining 3,087 HMM families formed targets in the current study. Additionally, some prediction methods required protein sequences as input; therefore first protein sequence was selected from the seed sequences as a representative sequence for each of these 3,087 DUF families as query.

### Computational methods used for remote similarity detection

Five different computational approaches were employed to recognize relationships involving these difficult families. These distant relationship detection methods require either sequences or profiles of the families as inputs and potentially aid in structural annotations. For each of these methods details about the inputs, search databases, search parameters and detection of unambiguous hits leading to recognition of relationships are described below.

#### SCOP-NrichD database

Three thousand eighty seven HMM models were queried against SCOP-NrichD database [23] using hmmsearch [28]. Additionally, representative sequences for 3,087 DUF families were also queried against SCOP-NrichD database using jackhammer for 5 iterations [28]. Hits with E-value for full sequence ≤ 0.001 and at least 60 % query length coverage were considered as acceptable hits for both hmmsearch and jackhammer searches. It had been observed previously that due to profile drifting, few related sequences may be missed in searches in SCOP-NrichD database and therefore searches were also made in natural sequence database (SCOP-DB) [23] and the all hits were pooled together for further analysis. While identifying remote homologues, additional areas of concern were that the pooled hits may belong to different families, superfamilies or even folds. If predictions belong to different domain regions, both predictions were retained. However if the hits belong to the same region, then the following decision tree was implemented:

i. If all hits belonged to the same SCOP superfamily, then the superfamily of the hit with lowest E-value and maximum query length coverage was chosen.
ii. If the hits belonged to different SCOP superfamilies but same fold, then the number of hits from each superfamily were compared and the superfamily with highest frequency was selected.
iii. If the hits belonged to different SCOP folds or classes, then the superfamily with the highest number of hits was selected, if and only if the difference between the number of hits of the highest and the second highest SCOP superfamily was more than ten.

#### SUPFAM+ database

SUPFAM+ database clusters evolutionarily related Pfam families together with the help of their structural associations [24]. This database also provides a list of Pfam to SCOP superfamily associations derived from direct or indirect relationships. Briefly in SUPFAM+ database, 14,831 profiles from Pfam families were incorporated with secondary structure and hydrophobicity information and then queried against similar 3,901 profiles of SCOP families using AlignHUSH [29]. Hits with Z-scores ≥ 7.5 and 60 % query length coverage were regarded as remote homologues. Additionally, all Pfam profiles were also queried against a database of Pfam profiles to obtain a Pfam-Pfam mapping (search parameters: Z-score ≥ 9, 80 % query and hit length coverage). This mapping was used to obtain Pfam-SCOP indirect relationships. From these Pfam-SCOP mappings (direct or indirect), predictions for 3,087 DUF families were extracted. Ambiguous predictions in SUPFAM+ database were resolved using Z-scores and the number of hits for each SCOP superfamily. Firstly, if hits belonged to same superfamily, then SCOP superfamily with the highest Z-score was selected. Secondly, if hits belonged to different superfamilies but same fold, then the number of hits from each superfamily were compared and the superfamily with highest frequency was selected. Thirdly, if hits belonged to different folds but same class, then the Z-score difference between the highest and second highest should be ≥ 4 and lastly, if the hits belonged to different SCOP classes then the difference between the highest and second highest Z-score should be ≥ 6. Using these parameters, SUPFAM+ database provides 5,280 Pfam – SCOP superfamily mappings consisting of 5,002 direct mappings and 278 indirect mappings, from which predictions for DUF families were extracted.

#### Superfamily database

The SUPERFAMILY database consists of 15,438 hidden Markov models representing 2,019 proteins superfamilies with known structure [65]. Representative sequences from 3,087 DUF families were queried against this database (version 1.75) using hmmscan program of the HMMER3 suite of programs at an E-value threshold ≤ 0.001. The ambiguity was resolved using same protocol described for SCOP-NrichD database.

#### pDomTHREADER (fold recognition)

Each representative sequence of 3,087 DUF families was subjected to fold prediction by pDomTHREADER [26]. Hits with P-value ≤ 10^−5^ were considered for further analysis (*i.e.,* hits tagged as CERTAIN). PDB hits obtained from pDomTHREADER were then mapped to SCOP-extended database (v 2.03) [66] to identify the associated SCOP superfamily. For certain families, pDomTHREADER may predict multiple SCOP superfamilies for the same domain region. To resolve such ambiguities, SCOP superfamily with the highest frequency was chosen.

#### HHsearch

Multiple sequence alignments for 3,087 DUF families were converted to HHsearch compatible profiles (using reformat.pl in HH-suite of programs) [67] and were queried against a library of 19,247 Hidden Markov Models (HMM) (downloaded from HH-suite ftp site), representing all the structures in SCOP95 (ASTRAL release 1.75, filtered for 95 % sequence identity) [27]. An E-value threshold of 0.001 was used to identify homologues. To resolve uncertainty amongst hits, decision tree describe above for SCOP-NrichD database method was followed.

### Removing ambiguous cases for all methods combined

For each DUF, predictions could be by all, any four, any three, any two or by a single method. While combining the structural annotations by these methods, any possible ambiguity was dealt with care. Firstly, overlaps in domain boundaries were checked. A domain overlap was considered if the domain boundaries by two different methods have at least 60 % length coverage of any one of the domains. Then the associated SCOP superfamilies for the each overlapping domain region were compared and ambiguous cases were removed. If ambiguity exists between any two methods, the detected relationship was excluded from the list. However if a case existed for a DUF family, where four out of five methods recognized the same SCOP-superfamily and one method recognized differently; such cases were included.

### Test dataset for evaluation of the approach employed

For the purpose of evaluation of the five methods employed, a test dataset of Pfam families with known structure and SCOP domain information was generated. Using the PDB ↔ Pfam mapping provided by the Pfam database, 699 DUF families were found to have at least one member from each family with known structure. 398 DUFs families amongst these also had defined SCOP domains in SCOP-extended database (SCOPe v2.03 [66]). Queries from these DUF families were used to test the success rate, precision and error rates of the 5 methods used.

### Assessment of computational methods for detection of distant protein similarities

To assess the performance of the 5 methods used in this study, profiles or representative sequences of 398 DUF families with known structures and SCOP domain definitions were queried against their respective databases. SCOP fold assignment for each query was assigned using the same search parameters as defined above for each method. For each query, success rate, precision and error rates were computed. Hits from the same SCOP fold as that of known structure was considered as correct fold assignments and hits belonging to different SCOP folds were regarded as wrong fold assignment. Thus, success was considered, if the predicted SCOP fold was same as the SCOP fold of the known structure. For the purpose of assessment the following definitions were employed:

$$ Success\  rate=\left(\frac{TP}{N_{total}}\right)\times 100 $$

$$ Precision=\left(\frac{TP}{TP+FP}\right)\times 100 $$

$$ Error\  rate=\left(\frac{FP}{TP+FP}\right)\times 100 $$

Where, TP = Number of correct fold assignments

FP = Number of wrong fold assignments

N_*total*_ = Total number of queries

### Phylogenetic information for DUF families

Phylogenetic membership of Pfam families was determined by using the strain-specific taxonomic sequence identifier (provided by UniProt [68]) for each sequence in that family. Each strain was then mapped to the taxonomic phylum using the NCBI taxonomy (ftp://ftp.ncbi.nlm.nih/pub/taxonomy/) [69]. Thus, for each DUF family, we enlisted the sequence identifiers for all members of the family and extracted their kingdom and phylum information using the NCBI taxonomy and report all the unique kingdoms for that Pfam family.

### Structural domain and GO annotations

Database of domain-centric Gene Ontology (DcGO) is a comprehensive ontology database for protein domains [54]. It provides associated ontological terms (Molecular function, Cellular process and biological process) for SCOP superfamily and families. GO terms with high information content (IC ≥ 1.5) were extracted from this database for each predicted SCOP superfamily for the query DUF family.

### Structural domains and Enzyme classification.

A list of 33,843 enzymes with associated EC numbers was retrieved from Protein Data Bank [70], from which all single domain proteins with SCOP domain definitions were extracted. Using these structure - function relationships, a list of associated EC number for each SCOP superfamily was generated. This SCOP superfamily – EC mapping was used in determining the associated enzyme reactions with each SCOP superfamily.

### Model building and refinement for DUF3050, DUF1572, DUF2092 and DUF1636

For homology modeling, template structure was selected based on the remote similarity detection by various methods *i.e.* the closest structure reported by the top hit of all the prediction methods was chosen as template for model building. Promals3D [71] was used to generate accurate alignment between the target and the template. Models were built using MODELLER [72] with high confidence secondary structure constraints derived from PSIPRED [73] prediction, followed by loop optimization. The model with lowest DOPE score and allowed backbone angles was refined and minimized using Rosetta minimization program [74].

### Assessments using online sequence search tools and datasets

In addition to the five very sensitive search methods and databases employed here, we also submitted the seed sequences of DUF families that were associated with a structural template in SCOP-NrichD database searches to the Batch web CD-search tool against all the available search databases [32]. We also submitted a representative sequence from each of the seed sequences of the DUF families to MESSA, a meta-server that integrates various tools for structure and function prediction [31].

## Reviewers’ reports

### Reviewer number: 1 First report (Eugene Koonin):

(1) Mudgal and colleagues describe a systematic effort on predicting the structures and functions of the DUFs in the Pfam database. This is a laudable, indeed, an important goal, and the authors use a battery of appropriate, sensitive and powerful methods. The number of resulting predictions is quite impressive.

*Authors’ response: We thank the reviewer for his appreciation.*

(2) My considerable disappointment, however, is with the “highlights” chosen by the authors to illustrate their success. Each of the three highlights is, to put it bluntly, quite trivial. The homologies reported by the authors are certainly valid, and each is easily detected by NCBI CDD search with low e-values. So to identify the provenance of these DUFs, the panel of sophisticated methods applied by the authors was unnecessary.

*Authors’ response: Thanks very much for bringing this important point up for discussion. That the homologies of the three examples we had selected could be detected in the NCBI CDD search, with low e-values, was indeed an oversight. We have now analysed several cases of distant similarity detection by five methods (presented in Table S3) to identify ‘non-trivial’ examples that are not easily detected by standard search procedures. We have now included a summary of our observations for 39 potential enzymes in our dataset, that were recognized by searches in the SCOP-NrichD database, in a new table (in Additional file 6: Table S5). To support our findings, we have also included the nature of hits, if any, when the members of the DUF families were queried in CDD searches and summarised the results of searches of these queries in meta-servers such as MESSA in the same table. In doing so, we hope to present the non-trivial nature of annotation of some of the results.*

*We find that of the 39 cases listed in the Table, 20 DUFs do not identify any other domain in CDD searches, 10 DUFs identify domain profiles at low confidence (<0.00001). 7 DUF families find hits at high confidence, which agree with the hits reported in this study. Detailed analysis of the cases that were ‘non-trivial’ showed that 12 examples are associated with the β-propeller fold, which is characterised by a repetitive structural unit, while seven families are associated with the P-loop NTP hydrolase and triose phosphate isomerase folds that are themselves associated with a very wide range of functions. Indeed, in the entire dataset of the 614 families with hits from multiple approaches, only 145 families were connected to a fold seen in an enzyme. Another difficulty in the annotation of these proteins is that many of the DUFs are short sequences (21 families are <100 residues long: in Table S5). An underlying assumption in such annotation efforts is the globular nature of the protein; however, if they constitute short sequence segments that are either disordered or parts of a larger complex, then the annotation of such cases based on known protein domain families is even more challenging. A new section titled “Short length of domains, repeats and domain definitions can limit the scope of function recognition” has now been included to discuss these examples.*

(3) Moreover, DUF1636 and DUF2092 are already annotated in many genomes as a metal-binding protein and a periplasmic protein, respectively. Not very precise annotation but each points in the right direction. So these are not impressive highlights. Furthermore, the most common results of “de-DUFing” included identification of repetitive domains such as ARM, TPR, beta-propellers etc. This is useful but not particularly informative when it comes to the prediction of the functions of the respective proteins.

*Authors’ response: We acknowledge that these examples are already annotated in many genomes with the associated function. Our response is that large-scale application of various methods can point us to a likely functional role. However, the confidence in the assignment and the detailed analysis of sequence and structural signatures of the members of each family merits a close examination of the proposed relationship. Indeed, for many of the cases that we have studied, either structure is preserved or functional detailing is altered and ‘classical’ sequence/ structural signatures of the associated function or fold are not always conserved. We discuss in detail the two new examples of DUF3050 and DUF2071 in the section on ‘Highlights’ and ‘Enormous divergence and mutation of functional residues during evolution’.*

*Having said this, although ‘trivial’ in the light of earlier reports of their potential function, we would like to retain the examples firstly because the use of a consensus approach to deduce structure and function therein, for protein families of unknown function, improves the confidence in prediction. Secondly, we wanted to examine the connections at a greater resolution, not merely suggest the link. Therefore, more detailed assessment of structural and functional features was performed to explore if these signatures are conserved. We anticipate that for the examples discussed here the quality of the detailed analysis is larger and better than the quality of the inputs on which we have no influence. We believe that for the three examples already described here, such a level of analysis has been performed and reported fairly convincingly, and that it might interest the readers.*

(4) All these observations speak more to the poor state of the current annotation of the available protein sequences than to the power and potential of the methods employed by the authors. So I think the paper could be considerably improved if the authors could present examples of actual non-trivial discoveries that do not easily come up in standard BLAST (which includes a CDD search).

*Authors’ response: We agree that annotation quality and pace dictate the quality of the input datasets and the detected relationships. However, with improved confidence in the pointers to function, such as through the computational structural association described here, we hope that protein function annotation is also improved.*

*We agree that these searches could have been easily carried out and have now included results from these searches explicitly in (Additional file 6: Table S5).*

(5) A minor but certainly vexing issue with terminology: “remote homology detection” is not legitimate usage. It should be “remote similarity” or perhaps “detection of homology on the basis of remote similarity”.

*Authors’ response: We have corrected usage of the term remote homology detection and replaced it with the suggestions of the reviewer.*

(6) Quality of written English: Needs some language corrections before being published

*Authors’ response: We have made language corrections and rephrased several sentences and also checked the manuscript for grammatical errors.*

### Review 2: First report: Dr. Frank Eisenhaber:

(1) The assignment of biological functions to yet uncharacterized regions of the genome is if not the most urgent task in life sciences and every progress in this direction will further the advance in biomedical and biotech applications (e.g., JBCB 2012 v5, 1271001). Thus, a periodic re-visiting of sequences without functional annotations is a worthwhile research investment fuelled by the hope that larger sequence databases or more modern, more sensitive methods might find new hints. The work of Mudgal et al. tries a battery of five approaches and finds new, valuable functional and structural hints for potentially globular segments in 614 domains of previously unknown function. Three of the cases with especially rich annotation are discussed in great detail.

*Authors’ response: We thank the reviewer for his positive reception of our work and appreciation of the detailed functional annotation presented here. We also thank the reviewer for drawing our attention to the recent review on extent of functional annotation and limitations in gene-function annotation, which we found very useful and have cited in the background section.*

(2) It should be noted that the DUFs were generated essentially by criteria of sequence similarity among sequence segments of various origins. Not necessarily they do represent globular segments for which homology approaches are suitable for functional annotation (e.g., Wong et al. BMC Bioinformatics 2014, 15:166; Biol Direct. 2011 6:57 and PLoS Comput Biol. 2010, 6:e1000867) although many of them are likely globular. This could be explored with tools checking for sequence complexity, intrinsically disordered structure, etc.

*Authors’ response: We agree with the reviewer’s points and found the mentioned papers relevant to our understanding of some of the difficulties faced in annotating the DUFs and have therefore cited them in section on - Short length of domains, repeats and domain definitions can limit the scope of function recognition. Thanks very much for pointing them out to us.*

### Review 3: First report: Dr. SriKrishna Subramanian:

(1) In this manuscript entitled, “De-DUFing the DUFs: Deciphering distant evolutionary relationships of Domains of Unknown Function using sensitive remote homology detection methods”, Mudgal et al., use five different approaches to map DUFs with no known three-dimensional structures to SCOP superfamilies and use this information to further predict probable functions. The combination of different approaches as compared to single methods to predict structure and function have been used rather successfully in the past (for example metaservers are top predictors in the CASP competition). Overall the paper is scientifically sound.

*Authors’ response: We thank the reviewer for his kind comments that the search methods and evaluations carried out here are scientifically sound.*

(2) The authors have employed different methods to predict structure and function for 3,087 DUFs, but they have not compared their results to the DUFs, which have already been grouped with other families into a single Pfam clan. The functions and structures for such DUFs may be predicted with relatively high confidence based on other families in the same clan and the general function of the clan as a whole. The authors should include the statistics of DUFs other than the ones already grouped under known Pfam clans i.e., those predicted only by their methods.

*Authors’ response: Clan information and the grouping of Pfam profiles into such groups is an indicator of the functional roles of a domain family through association of the any of the members with a protein of known structure/ biochemical role. Of the 3,087 DUF domain families, 2,766 do not have any clan information and therefore do not contain any annotation. Since this is an approach that attempts to integrate annotations obtained from more than one search method, we chose to examine only those annotations that are reported at high confidence through the multitude of methods employed here and therefore report only 614 of the domain family annotations that could be annotated through any one of the approaches. Of the 614, 69 % of the families do not have Pfam clan associations (Table S4, Figure S1). 142 of these families could be associated with a SCOP fold using NrichD database searches alone, with 52 of these families implied to have an enzymatic role. Of the remaining annotations with Pfam Clan associations, SCOP-superfamily recognitions with high confidence were compared and they also concur.*

(3) The study compares the output of five methods and provides an idea of the reliability based on consensus of these methods. It appears that only those predictions, which are high scoring in each of these methods, are used and listed on the online database. However, it would be of interest to the community if results for all DUFs (even low scoring) are provided in the online database with a clear warning. Further, it would be good to have this updated at regular intervals.

*Authors response: It is true that hits that qualify stringent filters alone are reported for each of the methods. Since this is a large-scale analysis of 3,087 domain families, we set E-value filters at the run time of the program. Hits with low confidence would therefore, not be reported. However, alignment coverage criteria failed for many hits despite good E-values. As suggested by the reviewer, we have provided all result file, including low-confidence hits, in the following link (**http://proline.biochem.iisc.ernet.in/RHD_DUFS/ALL_RESULTS**). Appropriate links are provided to the results files in each folder. A link to the poor-confidence data with appropriate warnings has been made in the web resource. Although we had not intended that the web resource with links to the hits be considered a database, we will consider updating the predictions and the result file at appropriate frequency commensurate with update of the search datasets that have been employed here, such as the SCOP-NrichD database etc.*

(4) It appears that the function prediction is carried out by HMM searches of the DUFs against other Pfam families. So why are the authors restricting themselves to DUFs for which no structural information is available. They could very well extend this to all DUFs.

*Authors’ response: Thanks for bringing this point up. We would like to clarify that here we have carried out HMM predictions against databases derived using structural information. We have not performed HMM searches of DUFs against other Pfam families since the emphasis here was to first associate a structure with reasonable confidence and then attempt functional annotation. Although SUPFAM+ database generation involves Pfam-Pfam associations, which are used to derive indirect relationships, these are not employed to searches performed using other 4 methods.*

*We have now provided the predictions for the 699 DUF families for which structural information is available in Additional file 2: Table S2. Of the 699, 398 have a member with structure and associated SCOP fold, the remaining members are not yet associated with a SCOP fold. SCOP fold associations match for 98.9 % of the 398 folds with four false positives in the pDomTHREADER and SUPFAM+ database searches. It is also heartening to note that a common prediction is not obtained for only six of the 699 DUF families in the dataset showing that each of the five methods demonstrates a low false positive rate in the cases of ‘known structure’. Since the 699 DUF families were employed in deciding the search conditions to achieve high specificity and sensitivity when using the multiple approaches and they were already associated with a structure that could indicate function, we performed the analysis on the remaining 3,087 domain families.*

(5) The authors do not compare their results with the prediction results with any existing Meta-servers (Eg. MESSA, metabasic etc., which are top CASP predictors) that combine various tools for sequence and structure comparison and structure and function prediction.

*Authors’ response: We thank the reviewer for suggesting that we could apply a meta-server to compare our results with. We employed MESSA for evaluating the nature of hits for 39 of the potential enzyme families identified in SCOP-NrichD database searches. Since the server integrates multiple tools and reports hits, we chose to use a single representative query for each of the DUF families. It must be mentioned here that the server does not send reports of the results after the entire run and the user has to keep the window indicating the job-run alive, to view the results. This was either because the server running the program failed locally such as the STRING searches or no convincing results were obtained. Although we have compiled the results and reported them in (Additional file 6: Table S5), it was quite cumbersome to collate these findings. If one were to perform this for every member of each DUF family, this would be time consuming and also tie down the analysis.*

*In our opinion integrated web-servers work best for analysis involving one or few protein sequences of interest to the user.*

(6) The authors have used SCOP-NrichD and describe it as the first large-scale implementation of artificially enriched sequence database to detect remote homology. It would be worth comparing and discussing the DUFs that are reliably detected by other methods and not by SCOP-NrichD.

*Authors’ response: This manuscript describes the first large-scale implementation of SCOP-NrichD database in detecting distant evolutionary relationships. The number of unique hits that are identified by the individual methods is included in Figure**1**and in the section on “Performance of various methods employed in the study”. The section on “Distribution of folds and superfamilies” describes the limitations of designed sequences in orphan folds over other methods and advantages of other methods such as SUPFAM+ database that uses indirect relationships as well.*

### Minor concerns

1. Reference to prior literature is missing at many places in the manuscript. For example

(i) where the authors mention “In our study as well, predictions for DUF354 family....” authors do not refer to any previous study.

*The DUF354 predictions are consolidated findings of the five methods employed here and not reported elsewhere. We have clarified this point.*

(ii) In the methods section authors have provided reference only in the SCOP-NrichD sub-section of the five approaches that they have employed but not for others. Even though the references have been provide in the main text, it is desirable that proper referencing be done independently for the methods section.

*Author’s response: We have included the references appropriately.*

2. In the methods section, the authors do not describe the tools used to construct the hmm profile from the multiple sequence alignment for the HHsearch.

*Author’s response: We have described this in the method’s section.*

3. The figure legend numbering and description does not match the serial order of the figures. The “Figure 6” in the legends describes Figure 2.

*Author’s response: Thanks for pointing this out. We have now corrected this.*

4. In Figure 2 there is a spelling mistake in “Multidrug efflux transpoter AcrB” which should read as “Multidrug efflux transporter AcrB”.

*Author’s response: Thanks for pointing this out. We have now corrected this.*

## Additional files

Additional file 1: Table S1. Success rate, precision and error rates for 398 DUF families of known structure using the five remote similarity detection methods. (PDF 48 kb) Additional file 2: Table S2. SCOP Superfamily recognition for 699 DUF families with known structures for at least one member of the family. 398 of these also have an assigned SCOP domain superfamily. (XLSX 91 kb) Additional file 3: Table S3. SCOP Superfamily recognition for 614 DUF families using 5 different computational methods. (XLSX 156 kb) Additional file 4: Table S4. SCOP Superfamily recognition for 423 DUF families which are not associated to any Pfam Clan. (XLSX 118 kb) Additional file 5: Figure S1. Venn diagram representing the distant relationship recognition by 5 methods for 423 DUFs, which are not associated with any Pfam Clan. (PDF 631 kb) Additional file 6: Table S5. SCOP superfamily recognition for DUF families using SCOP-NrichD database searches. (XLSX 39 kb) Additional file 7: Table S6. Prediction details by different methods for ambiguous predictions. (XLSX 14 kb) Additional file 8: Figure S2. Schematic representation of full-length protein sequences consisting of DUF659 domain. The domain boundaries for the N-terminal Zinc binding domain, DUF5669, C-terminal dimerization domain and the Ribonuclease H-like domain were derived from InterPro database and searches in SCOP-NrichD and SUPERFAMILY database. (PDF 1595 kb)

## Abbreviations

DUF: Domain of unknown function
UPF: Uncharacterized protein family
PSSM: Position specific scoring matrix
HMM: Hidden Markov model
