# Supplementary material for: De-DUFing the DUFs: Deciphering distant evolutionary relationships of Domains of Unknown Function using sensitive homology detection methods
Source: Biol Direct. 2015 Jul 31;10:38. doi: 10.1186/s13062-015-0069-2 (PMC4520260; doi:10.1186/s13062-015-0069-2)
Supplement: Additional file 1: Table S1. — Success rate, precision and error rates for 398 DUF families of known structure using the five remote similarity detection methods. (PDF 48 kb) [file 13062_2015_69_MOESM1_ESM.pdf]

Table S1. Success rate, precision and error rates of five computational methods for remote homology detection.

	**SCOP-NrichD database**	**SUPFAM+ database**	**SUPERFAMILY database**	**pDomTHREADER**	**HHsearch**
True positives	365	256	369	236	373
False positives	0	2	1	1	0
Success rate/ Sensitivity	91.70%	64.32%	92.71%	59.29%	93.71%
Precision	100%	99.23%	99.73 %	99.57 %	100 %
Error rate	0 %	0.77 %	0.27 %	0.43 %	0 %
