# Supplementary figures and images for: De-DUFing the DUFs: Deciphering distant evolutionary relationships of Domains of Unknown Function using sensitive homology detection methods
Source: Biol Direct. 2015 Jul 31;10:38. doi: 10.1186/s13062-015-0069-2 (PMC4520260; doi:10.1186/s13062-015-0069-2)

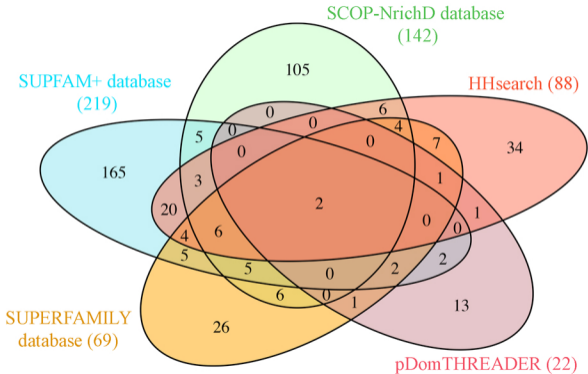

Supplement: Additional file 5: Figure S1. — Venn diagram representing the distant relationship recognition by 5 methods for 423 DUFs, which are not associated with any Pfam Clan. (PDF 631 kb) [file 13062_2015_69_MOESM5_ESM.pdf]

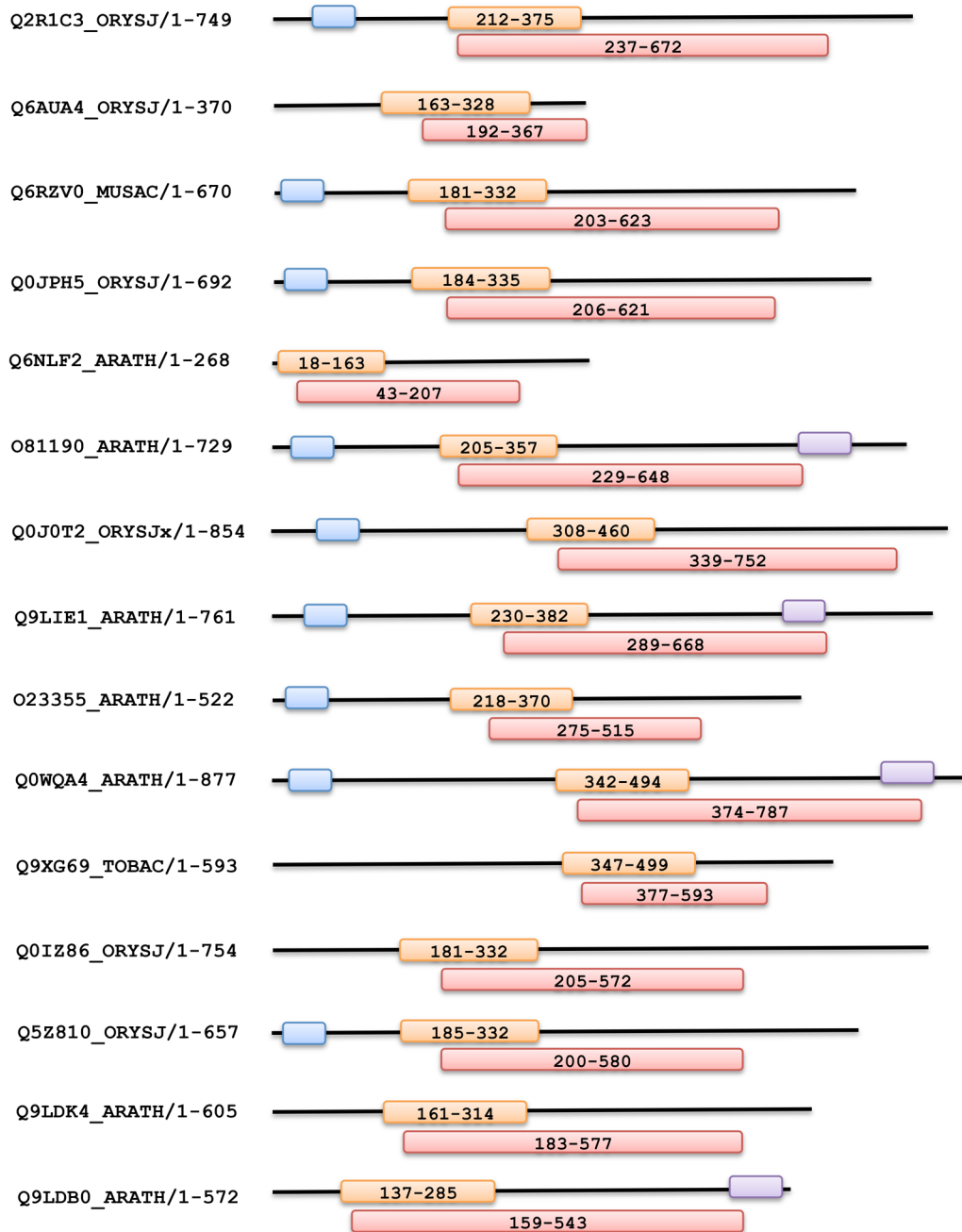

Zinc binding domain
  C-terminal dimerization region

DUF659
  Ribonuclease H-like (c.55.3)

Supplement: Additional file 8: Figure S2. — Schematic representation of full-length protein sequences consisting of DUF659 domain. The domain boundaries for the N-terminal Zinc binding domain, DUF5669, C-terminal dimerization domain and the Ribonuclease H-like domain were derived from InterPro database and searches in SCOP-NrichD and SUPERFAMILY database. (PDF 1595 kb) [file 13062_2015_69_MOESM8_ESM.pdf]
